# Significant risk of COVID-19 and related-hospitalization among patients with adrenal insufficiency: A large multinational survey

**DOI:** 10.3389/fendo.2022.1042119

**Published:** 2022-11-10

**Authors:** Christine Yedinak, Ian Louis Ross

**Affiliations:** ^1^Department of Neurosurgical Services, Oregon Health and Science University, Portland, OR, United States; ^2^Department of Medicine, University of Cape Town, Cape Town, South Africa

**Keywords:** adrenal insufficiency: incidence COVID-19 SARS-CoV2, COVID-19, adrenal insufficiency, incidence, hospitalization

## Abstract

**Objective:**

To determine self-reported incidence and potential risk factors for COVID-19 in patients with adrenal insufficiency (AI).

**Methods:**

A 27-item AI survey was developed for AI and COVID-19 status, vetted by specialists and patients, and distributed *via* social media, websites, and advocacy groups. Participation was voluntary and anonymous. Data were collected from September 20^th^, 2020 until December 31^st^, 2020.

**Results:**

Respondents (n=1291) with self-reported glucocorticoid treatment for AI, completed the survey, with 456 who reported having symptoms and were screened for COVID-19 during 2020; 40 tested positive (+ve), representing an 8.8% incidence. Of the COVID-19^+ve^, 31 were female (78%), with mean age of 39.9 years. COVID-19 among AI patients occurred most commonly in those aged 40–59 years (n=17; 42.5%); mean time since AI diagnosis was 13.5 years (range 0.2−42.0 years). Pulmonary disease, congenital adrenal hyperplasia, and higher maintenance doses of glucocorticoids were significantly associated with +ve COVID-19 (*p*=0.04, *p*=0.01, and *p*=0.001, respectively. In respondents the cumulative incidence of COVID-19^+ve^ during 2020 was 3.1%; greater than the 1.03% worldwide-incidence reported by WHO, by December 31^st^, 2020. There was a 3-fold (95% CI 2.16-3.98) greater relative risk (RR) of COVID-19 infection and a 23.8- fold (95% CI 20.7-31.2) RR of hospitalization in patients with AI, compared with the global population.

**Conclusion:**

A markedly raised RR of COVID-19 and hospitalization in respondents reporting chronic AI was detected. We found that a diagnosis of congenital adrenal hyperplasia, age>40 years, male gender, pulmonary disease, and higher maintenance doses of glucocorticoids were associated with greatest risk.

## Introduction

As of July, 2022, there had been > 555 million cases of coronavirus (COVID-19) worldwide. Cases continue to spike with a 7 day average worldwide peak mid-January 2022 of over 3.4 million cases (1, 2). Given the spectrum of presentation from asymptomatic illness to severe disease, variable availability or under-utilization of testing, this may be an underestimate across all populations. Infection, in steroid dependent patients with AI, is the primary driver of adrenal crises and high mortality risk (3–5). This risk has been compounded since 2020 by emotional distress, limited access to medical practitioners associated with pandemic restrictions and staff availability, heightening the need for patient disease knowledge and self- management (4, 6, 7). With the more recent emergence of highly contagious SARS CoV-2 variants such as Omicron and BA.5, coupled with the difficulty predicting future mutations, providing patients with data-derived information to demonstrate level of risk more clearly may be helpful to reinforce the need for ongoing protective behaviors and strategies to mitigate high risk of infection, COVID-19 complications, adrenal crisis and catastrophic outcomes.

Angiotensin-converting enzyme-2 (ACE2) suppression or dysregulation of ACE/ACE2 antagonistic relationship has been proposed as a mechanism for COVID-19 infection susceptibility or severity (8). Ciliated epithelial cells of the respiratory tract are first affected by inhaled virus. Spike proteins of the SARS-Cov-2 attach to ACE2 cell surface receptors and gain ingress into the cell, aided by proteases such as TMPRSS2 and other facilitators. On entry, the protective effects of ACE2 in affected cells are downregulated, allowing unopposed ACE activity (3, 8, 9). Viral replicates attach to ACE 2 receptors in the endothelium of the vasculature and multiple organs such as the hypothalamus, pituitary, thyroid, heart, kidneys, pancreas, testes, adrenals and intestines (3, 10). Older age, male sex, obesity, smoking, ethnicity and comorbidities (for example, diabetes mellitus) have been associated with COVID-19 severity and worse outcomes (10–13) in the general population.

At the onset of the pandemic, leading endocrine societies issued statements of concern for increased risk of COVID-19 infection for patients with known AI (14, 15) However, the impact in patients with pre-existing hypoadrenalism is largely based on known immune suppressive effects of glucocorticoids and assumed risk. The precise risk of COVID-19 infection in patients with AI compared with the general worldwide population and based on factors such as glucocorticoid dose, individual risk behaviors and comorbidities has not been quantified.

We hypothesized that the risk of COVID-19 may be altered by the presence of AI, independent of etiologies and comorbidities, potentially conferring a worse prognosis compared with the general population. We aimed to compare the incidence of COVID-19 infection in individuals with known AI with the worldwide incidence of COVID-19 in 2020 and provide some insight into concomitant diseases or factors that may increase risk. Five groups of patients were included: Primary AI (PAI), secondary AI (SAI), congenital adrenal hyperplasia (CAH), tertiary AI (TAI) patients (a diagnosis of AI with a minimum of 3 months use of long-term glucocorticoid treatment for other than pituitary or adrenal etiologies) and other etiologies (*e.g.* adrenoleukodystrophy). We assessed COVID-19 symptom frequency, testing and diagnosis, age, concomitant disease, glucocorticoid dose, stress dosing, avoidance behaviors, and lingering symptoms on recovery among patients with AI.

## Materials and methods

### Research ethics, study design, and participants

Survey methodology was selected to recruit the maximum number of respondents worldwide over a short period of time. Given the need for global reach and for maximizing data capture, a questionnaire was developed for electronic distribution.

A 27-item questionnaire, was constructed utilizing a web-based tool (Question Pro.com software Survey Analytics LLC, Austin, TX, US 2002). Closed ended (Yes/No or multiple choice) questions, skip logic (conditional branching) and enforced answer techniques were used to minimize question misinterpretation, survey completion time and survey fatigue, and to eliminate missing data respectively. Items that required clarification, such as glucocorticoid type and dose, year of diagnosis, were also used to support the self-reported diagnosis of AI as a qualification for survey participation. (Appendix 1).

A panel of endocrine nurses, physicians, and patients from the Adrenal COVID Task (ACT) force (representatives from: National Addison’s Self Help Group, United Leukodystrophy Foundation, Living with CAH, and the World Alliance of Pituitary Organizations), reviewed and piloted the questionnaire for face content (objective consistency), internal and external validity. Questions were modified as needed and retested. Representatives adjudicating the questionnaire were from multiple countries, including the United Kingdom (UK), United States (US), Europe, Brazil, Australia, and South Africa. Questions were translated into 9 languages for review and publication.

The survey introduction and instructions informed the user of inclusion criteria (individual with AI or a family care-giver, nurse or physician caring for patients with AI) the voluntary nature of participation and anonymity measures. Consent was assumed with data entry. To progress through the questionnaire, participants qualified by adding diagnosis (etiology of AI), date of diagnosis, number of years of treatment, type and dose of glucocorticoids. This information also served to validate adrenal insufficiency status. An e-mail address of the principal researcher was provided. Institutional ethics review was waived due to the methodology and respondent anonymity.

An open survey methodology was utilized in publishing the questionnaire to allow broader accessibility. Internet access to the survey was distribution world-wide to endocrine medical teams, on social media, on mobile devices, on websites for national and international endocrine nursing societies and advocacy organizations also e-mailed members, including Adrenal Insufficiency United (AIU), the National Addison’s Disease Foundation (NADF), Adrenal Alternatives, Pituitary Foundation, Australian Addison’s Foundation, CARES Foundation, Magic Foundation, Addison’s Brazilian Association, the Australian Pituitary Foundation and World Alliance of Pituitary Organizations (WAPO). Global representation was sought to improve external validity.

Data were collected September 20^th^, 2020 - December 31^st^, 2020 and interrogated the period from January 1^st^, 2020 - December 31^st^, 2020. Therefore, some participants were asked to recall COVID-19 testing and symptoms from the early part of the year 2020. Given the high public profile and availability of information regarding SARs-CoV2 and COVID-19, memory of these events was assumed to be adequate. A single use identification number was used to anonymize data. Repeated access was exclusively permitted from the same internet protocol address for the purpose of completing a saved, but incomplete questionnaire. Some questions (*e.g.* zip or postal code) were optional and replaced by rural/urban, if the respondent had concerns regarding anonymity).

Questions covered domains of demography, comorbidities, hospitalizations, COVID- 19-related symptoms, self-protective behavior to limit COVID-19 infection, glucocorticoid dose, route of administration, availability of usual glucocorticoids, and COVID-19 screening and outcome. COVID-19^+ve^ respondents were additionally asked if they were treated at home or a hospital, if a stress dose of glucocorticoids was administered, if their glucocorticoid dose was altered during 2020, and if any residual symptoms occurred after their presumed recovery.

## Statistics

The dataset was analyzed by group. Group 1: respondents not tested for COVID-19, group 2: respondents who tested negative (-ve) for COVID-19, and group 3: respondents who tested positive (+ve) for COVID-19. Analyses were performed using SPSS27 (Version 27.0. Armonk, NY: IBM Corp) including descriptive statistics, crosstabs, where appropriate, Chi Squared to determine relationships between categorical variables, and ANOVA with *post hoc* Bonferroni correction, to assess the relationship between groups and continuous variables. Multinomial regression analyses were used to predict the influence of multiple independent variables, including symptoms and concomitant diseases on group outcomes. Independent T- tests were undertaken to assess relationships between continuous dependent variables and independent categorical variables and independent samples proportions to compare the proportions in two unrelated groups. A 2x2 contingency table was used to compare risk ratios for global and survey COVID-19 incidence and hospitalizations. Significance was accepted at *p* ≤ 0.05.

Relative risk (RR) of COVID-19 infection and hospitalization were calculated using a 2x2 table (where cumulative incidence in the COVID-19 group [40/1291] and cumulative incidence in a global group [80,611,600 infected/7.8 billion world population]) and the following formula to calculate a 95% confidence interval (CI):


$$\text{ln}\left(RR\right)\pm Z_{critical}\sqrt{\frac{1-{\hat{P}}_{1}}{n_{1}{\hat{P}}_{1}}+\frac{1-{\hat{P}}_{2}}{n_{2}{\hat{P}}_{2}}}$$

Ln = natural log; *P*1 = Cumulative Incidence in target population; *P*2 = Cumulative Incidence in global population; n1*P*1 = Number of positive cases in target population; n2*P*2 = Number of positive cases in global population (1, 2).

## Results

**Figure 1:**
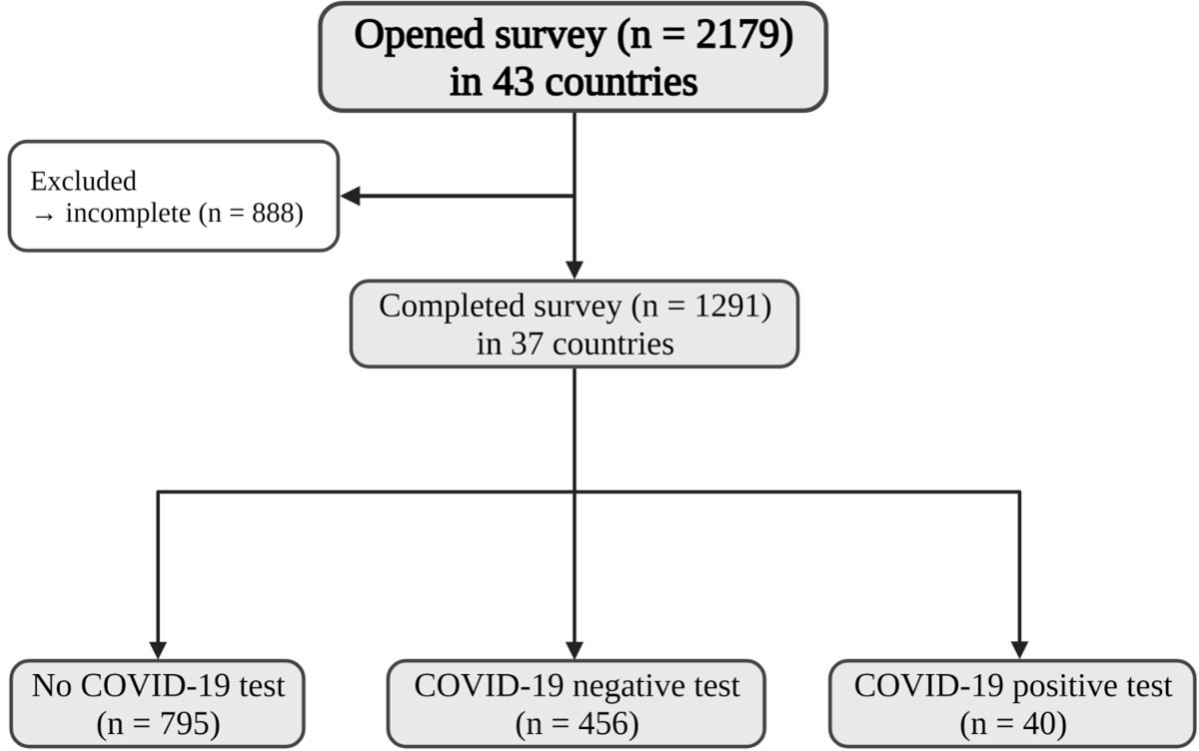
Flow Chart − Survey respondent inclusion criteria and COVID-19 status.

The survey was viewed 2179 times and opened 2013 times by individuals from 43 countries, with 1291 completions from 37 countries. (**Figure 1**). Access was permitted to complete an incomplete survey per single Internet Protocol (IP) address. Incomplete surveys accounted for 302 duplicate access attempts from the same IP address without data entry and were deleted. In addition, 101 attempts failed Captcha (excluding robot access) and 19 were for test access and were excluded. The remaining 300 who accessed the survey universally withdrew after capture of referral source and country of origin and prior to any other data entry. Survey completion rate was 81.1%. Surveys were completed by individuals with AI (87.5%), a family member for pediatric cases (12%), and by medical professionals (0.5%). Overall, mean age of subjects was 47.3 years (standard deviation; SD 18.3 years, range 0.25−81.0 years) with 81.3% female. Males were younger than females 43.1 years versus 48.3 years, *p* = 0.001.

Group 1, not tested for COVID-19 (n = 795) constituted 61.6%, group 2 COVID-19^-ve^ (n = 456) 35.3%, and group 3 COVID-19^+ve^ (n = 40) 3.1%, respectively (**Figure 1**).

Self-reported ethnicity was Caucasian in 931 (72.1%), European (*n* = 289; 22.4%) Brazilian and/or Hispanic (*n* = 27; 2.1%) and the remainder 3.3% (*n* = 43) comprising African, African-American, Asian, mixed ancestry, and any other ethnic group. More respondents identified as Brazilian/Hispanic were found to be COVID-19^+ve^ than Caucasian/European respondents (25.9% vs 2.6%, *p* = 0.001) (**Table 1**).

**Table 1:** Baseline demographic characteristics of responders (* *p*-value < 0.05 considered significant).

	Adrenal insufficiency (AI) aetiology					
	Primary AI	Secondary AI	Congenital adrenalhyperplasia	Tertiary AI	Other	*p-value
Respondent (n)	724	369	72	70	56	_
Sex: Female/Male (*n*)	590/134	294/74	55/17	61/9	49/7	0.3
Mean age; years (SD)	49.4 (17.1)	47.5 (17.3)	23.8 (17.8)	47.9 (19.2)	48.2 (18.8)	**0.01**
Mean AI duration, years (SD)	12.2 (11.3)	8.1 (8.9)	19.8 (15.3)	7.8 (6.5)	8.9 (9.1)	**0.048**
**Ethnicity (n)**						**0.003**
Caucasian	514	259	59	55	44	_
European	183	84	5	10	8	_
Hispanic	8	11	3	2	3	_
Other	19	15	5	3	1	_
**Protective Behavior; *n* = 1276 (%)**						0.4
Low risk	49.6	51.9	50	47.1	39.3	_
Moderate risk	40.1	40.3	40.4	40.6	50	_
High risk	8.1	7.7	6.9	10.1	10.7	_
**Mean hydrocortisone dose; mg (SD)**	24.1 (12.7)	21.1 (8.2)	19.56 (11.1)	26.2 (13.3)	21.7 (10.0)	**0.002**
**Country of Origin (*n*)**						**Total**
Great Britain	399	139	25	33	16	612
USA	175	166	36	28	23	428
Australia	40	9	0	1	4	54
Germany	30	12	0	2	4	48
Canada	17	11	2	2	5	37
Ireland	17	10	0	1	1	29
Sweden	15	2	0	0	0	17
Brazil	6	4	7	1	1	19
Spain	6	0	0	0	0	6
Italy	3	0	0	0	0	3
Netherlands	2	3	0	0	0	5
South Africa	2	2	0	0	0	4
Other	16	9	2	0	2	29

Bolded p values indicate significance.

Respondents’ diagnoses included PAI in 56.1% (*n* = 724), SAI 28.6% (*n* = 369), CAH 5.6% (*n* = 72), TAI 5.4% (*n* = 70), and other diagnoses 4.3% (n = 56). CAH respondents were significantly younger 23.8 years (*p* = 0.001) than all other diagnoses. There was a higher proportion of males with CAH. Overall, sex did not differ significantly between diagnoses (**Table 1**).

The mean age of COVID-19^+ve^ respondents was younger (39.9 years) versus those not tested (48.2 years) or COVID-19^-ve^ respondents (46.5 years) (*p* = 0.03) (**Table 2**). However, for the 40 COVID-19^+ve^, when divided into quartiles based on age (0–19, 20–39, 40–59, ≥ 60 years) the group 40–59 years (n =17; 42.5%) had the highest COVID-19 risk (*p* = 0.016).

**Table 2:** Respondent Characteristics with Respect to COVID-19 Test Results (*p-value< 0.05 considered significant; NS= not significant).

	Testing Status				
	None	Negative	Positive	% Total	*p-value
Respondent (n)	795	456	40		
Mean age, years (SD)	48.2 (19.1)	46.5 (16.6)	39.9 (18.3)		**0.03**
**Sex**					0.3
Female (n)	636	382	31	3	–
Male (n)	158	75	9	3.7	0.06
**Adrenal insufficiency (AI) aetiology**					**0.001**
Primary AI *(n* = 724)	449	256	19	2.6	–
Secondary AI *(n* = 369)	224	136	9	2.4	–
Congenital adrenal hyperplasia *(n* = 72)	46	18	8	11.1	0**.001**
Tertiary AI (*n =* 70)	33	33	4	5.7	–
Other (*n* = 56)	42	14	0	0	–
Mean symptoms; n (SD)	2.6 (2.5)	3.7 (3.1)	5.3 (3.4)		**0.001**
Mean AI duration; years (SD)	12.1 (11.3)	9.1 (9.6)	12.8 (9.8)		0.04
Mean hydrocortisone dose; mg (SD)	22.4 (11.6)	23.5 (9.8)	29 (21.0)		**0.001**
Missed doses (%)	0.034	0.05	0.125		**0.006**
Fludrocortisone (n)	321	466	4		0.5
**Ethnicity (n)**					0.5
Caucasian	558	340	25	2.7	0.5
European	190	92	7	2.4	0.7
Hispanic	19	12	7	18.4	**0.014**
Other	25	11	1	2.7	0.1
**Comorbidities (n)**					
Pulmonary	135	103	10		**0.04**
Cardiovascular	140	99	6		0.2
Diabetes	74	37	4		0.7
Gastric	44	39	4		0.09
Rheumatoid	50	43	2		0.1
Renal	33	20	2		0.9
Osteopenia/Osteoporosis	135	66	3		0.2
Pituitary Deficiencies	143	86	6		0.8
**Concomitant Comorbidities (n)**					0.5
none	255	138	17		
1	275	148	13		
2	153	89	5		
3	70	45	2		
> 4	50	37	3		

Bolded p values indicate significance.

Not surprisingly, COVID-19-related symptoms were more numerous for respondents with positive results, compared with groups 1 & 2 (5.3.vs 3.7 vs 2.6: p = 0.001). On multinomial regression analysis, respondents with a loss of sense of smell and those with fever were 3.2-fold (p = 0.001) and 2.1-fold (p = 0.04) more likely, respectively, to be COVID-19+ve than those either not tested or COVID-19-ve.

Of all the subgroups with AI who were COVID-19^+ve^, a diagnosis of PAI was reported in 47.5% (*n* = 19) of respondents, SAI in 22.5% (*n* = 9), CAH in 20% (*n* = 8) and TAI in 10% (n = 4) (**Table 2**).

Patients with CAH (72/1291) demonstrated a higher incidence of COVID-19, compared with other diagnoses [8/72 (11.1%): 6 females/2 males]; (*p* = 0.001). Respondents were largely female (55), older than males (26.1 years versus 16.4 years respectively [*p*=0.057]); (range newborn to 60 years), with an average of 19.4 years since diagnosis. The mean glucocorticoid dose was similar for males and females: mean 19.4 mg daily with 77.8% taking fludrocortisone versus 50% (4/8) of those reporting COVID ^+ve^ tests.

## Comorbidities

Pulmonary disease (asthma, bronchiectasis and pulmonary hypertension) occurred more frequently in respondents who were COVID-19^+ve^, compared with the remaining groups (*p =* 0.04). Although numerous other co-morbidities were reported, diabetes, hypertension and cardiovascular disease was not reported with greater frequency in the COVID-19^+ve^ group. (**Table 2**).

## Glucocorticoid replacement

Oral glucocorticoids were used by 97% of respondents; 2% used a continuous or pulsatile subcutaneous pump and 1% utilized subcutaneous injections. Hydrocortisone was used by most respondents (87.7%) and 77% of COVID-19^+ve^ patients. Mean duration of glucocorticoid replacement was 11.3 years (range 0.1−61.0 years). Daily dosage of glucocorticoids was reported in 1231/1291 (95.4%) respondents. All glucocorticoid doses were converted to hydrocortisone equivalent for comparison: mean (standard deviation; SD) daily dose of 23.02 mg (SD ± 11.5; range 0–150 mg daily). Intriguingly, some patients with SAI and TAI reported taking fludrocortisone (19/20 PAI [95%], 3/8 CAH, 1/9 SAI, and 1/3 TAI) (**Tables 1**, **2**).

Mean daily dose was different among the three groups; (*p*=0.001) and remained highest for the COVID19^+ve^ group (29 mg daily; SD ± 21 mg). Twenty patients used excessive replacement hydrocortisone equivalent doses (60-150 mg). In group 1, 3 respondents used 150 mg daily, and 6 respondents used between 60 mg and 100 mg daily (1.1%). In group 2, 8 respondents reported a maintenance dose of 60 mg −100 mg daily (1.8%). In group 3 (COVID- 19^+ve^) 3 respondents utilized between 80 mg and 100 mg daily (7.5%). After removing outliers from all three groups, there was no difference in mean dose among the groups (*p*=0.30). Duration of glucocorticoid replacement for AI was not associated with occurrence of COVID- 19 infection (*p*=1.0). Curiously, two patients noted doubling their regular dose at the start of the pandemic, but this could not be probed further (**Table 2**).

Pharmacy supply interruptions in 2020 resulted in 129/1291 (10%) of respondents changing maintenance doses and 56 (4.3%) missing doses for up to 3 weeks due to medication non-availability, postal delays, difficulty contacting an endocrinologist or refill refusal by a primary practitioner. In group 3 (COVID-19^+ve^) 5 respondents (12.5%) reported missing doses lasting from 2 days to 2 weeks and only 2 (5%) reported changing their usual glucocorticoids owing to supply interruptions. The majority 35/40 (87.5%) of COVID-19^+ve^ reported taking a stress dose (at least double) of steroids, with the onset of symptoms relating to COVID-19 (one person was asymptomatic) compared with either 70% not tested or 82.5% COVID-19^-ve^ (*p*=0.001).

## Prevention strategies for COVID-19

The majority (87%) of respondents across all the groups reported limiting SARS-CoV- 2 exposure by social distancing, social isolation or by wearing masks versus 9.1% who did not wear face masks in public. Exposure risk was divided into low, moderate and high-risk behavior for analysis, based on social isolation and mask wearing. In COVID-19^+ve^ respondents, 4 respondents (10%: 2 PAI, 1 SAI, and 1 CAH) indicated they engaged in high- risk behavior (**Table 2**).

## Relative risk of COVID-19 and hospitalization in patients with adrenal insufficiency

Overall, 3.1% of 1291 respondents tested COVID-19^+ve^. Persons per million SARS- COV-2^+ve^ by December 31^st^, 2020, numbered 10322 (1, 2), representing a 1.03% global annual cumulative incidence in 2020. The RR of infection for respondents in this cohort of AI was 3- fold higher than the world-wide incidence for the same time-period (95% CI=2.16-3.98-).

Of those COVID-19^+ve^ respondents, 75% reported receiving treatment at home, whereas 22.5% (9/40; 4 PAI, 5 SAI; 2 male, 7 female) were hospitalized for respiratory distress, and (5/9; 55.5%) reported pre-existing respiratory disorders. Based on a global population of 7.8 billion (December 31st 2020), with 80,511,600 reported COVID-19^+ve^ and 754,746 requiring hospitalization, the RR of hospitalization in our cohort was 23.78-fold higher than the global population (95% CI=20.69-31.24) (1, 16). ICU admission and ventilator support was reported by two females (5%) ages 51 and 54 years, one patient each from US and UK, respectively. Hospitalization was required for 4/16 COVID-19^+ve^ women of childbearing age with none reporting pregnancy.

## Residual symptoms after COVID-19 infection

**Figure 2:**
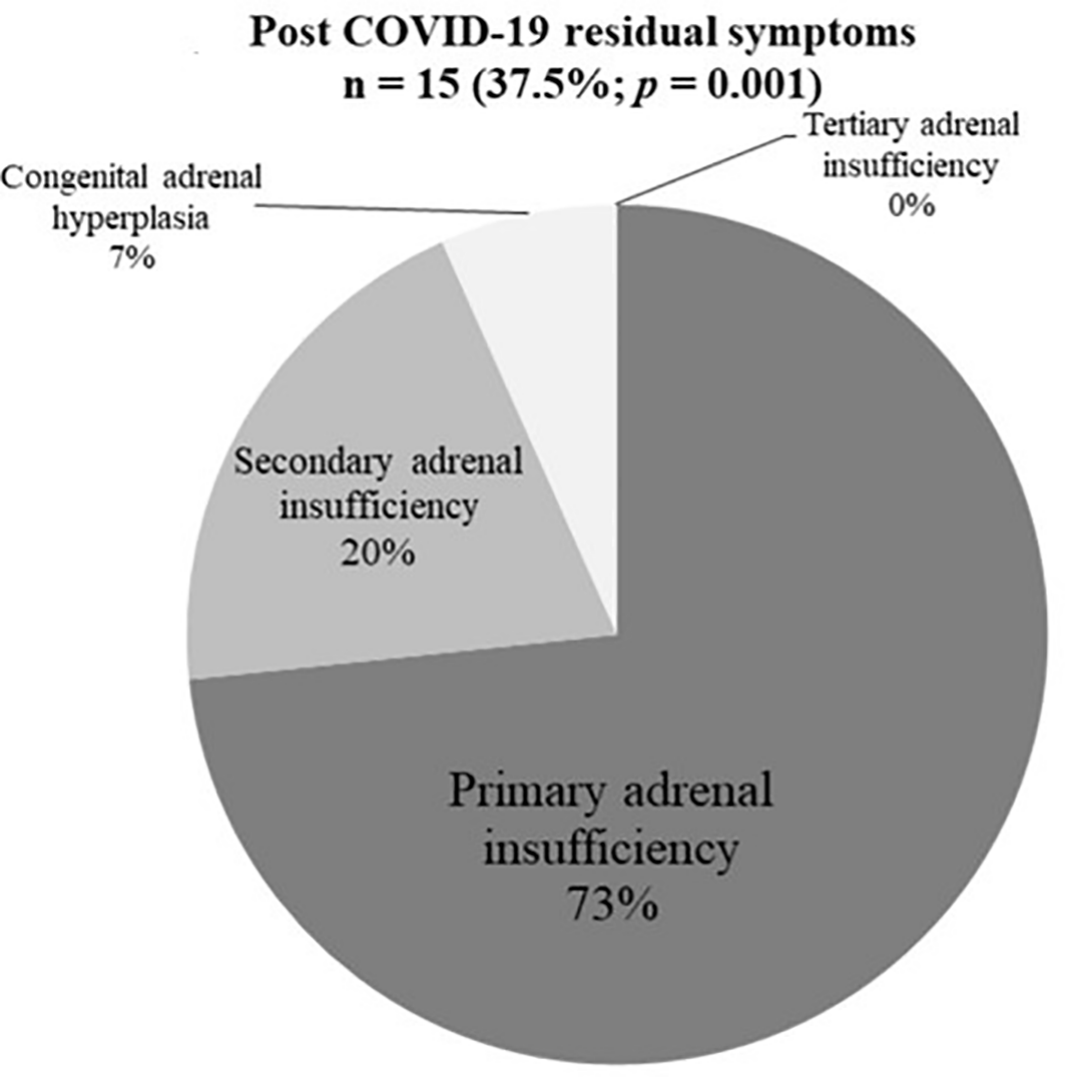
Post COVID-19 residual symptoms (n = 15).

Residual symptoms following COVID-19 infection were reported in 15/40 (37.5%) of respondents (**Figure 2**). SAI and CAH respondents reported residual symptoms more frequently than those with other diagnoses (PAI 3/20, SAI 6/9, CAH 5/8, TIA 1/3, *p*=0.02).

## Discussion

We demonstrated a higher cumulative incidence and RR of COVID-19 infection (3.1% versus 1.3%) and over 23-fold higher rate of hospitalization in patients with AI, compared with background incidence of COVID-19 in 2020. Patients with CAH had higher incidence of COVID-19 infection, with proportionately more males, compared with other AI diagnoses, but none with CAH required hospitalization. Age 40−60 years, pre-existing lung disease, and higher glucocorticoid doses were each independently associated with a higher risk of COVID- 19 infection. Increased symptom burden with loss of smell and fever, were predictors of COVID-19 infection. A greater proportion of COVID-19^+ve^ patients reported working outside the home without social distancing. Although all respondents recovered from COVID-19, 37.5% reported residual symptoms, particularly those with pre-existing lung disease and PAI.

Two studies reported low incidence of COVID-19 infection in patients with AI. A study from Lombardy, Italy, in early 2020 found no increased risk of developing COVID-19 among 279 patients with hypoadrenalism compared with the background population (17). Notably, only 12/279 patients reported COVID-19 testing.A second UK study found 2 COVID-19 ^+ve^ cases of 7 patients tested from a pool of 159 patients who were taking replacement steroids (16). These results are at odds with our study, in which we showed a two- fold risk of developing COVID-19. All studies depended on patient recall. In the Carosi study, COVID-19^+ve^ status was assumed based on patient recall of at least 2 symptoms considered highly indicative of COVID-19. Our study differed with respect to: larger sample size; longer duration; and data collected from a larger geographical area. In our study, all COVID-19^+ve^ patients reported testing. Stringent lockdown regulations and differential environmental viral loads as the pandemic progressed may also account for a variable risk of COVID-19. It is notable that the Omicron variant did not emerge until 2021 and therefore is not reflected in either study (18). Our data signals the necessity for careful mitigation strategies, acknowledging that patients with hypoadrenalism and COVID-19 may be at risk for a worse outcome.

An endogenous, stress induced increase in adrenal glucocorticoids in the context of infection is a key modulator of cytokine production and adaptive response inhibiting inflammation and helping to avert a cytokine storm (19, 20). However, prolonged critical illness, acute respiratory distress syndrome and sepsis are known to result in relative AI (glucocorticoid deficiency) and treatment with glucocorticoids is recommended (21). In patients with no history of AI requiring hospitalization with COVID-19, a high incidence of AI was found with lower cortisol levels associated with more severe illness (22). The Recovery trial demonstrated decreased COVID-19 related mortality risk and shorter hospitalization in non-AI patients hospitalized with respiratory distress or requiring mechanical ventilation (23, 24). No benefit of glucocorticoid treatment has been found in patients without respiratory compromise.

Long-term and/or high dose glucocorticoids are known to inhibit NK cell activity, resulting in lymphopenia, inhibition of macrophage differentiation and proliferation, and B and T-cell activation, potentially producing a more severe phenotype in AI (19, 25). Olnes et al. (25) demonstrated lymphocyte suppression and persistent NK cell deficiency after a single infusion of hydrocortisone in patients with normal adrenal function. NK cell deficiency has been demonstrated in patients with PAI compared to healthy controls (10, 26, 27). There is little data specific to SAI. Natural killer (NK) lymphocytes directly target antigens and influence B and T-cell responses, limiting viral spread and cell damage (26). As a result, patients with pre- existing AI are more likely to have increased susceptibility to COVID-19, a higher risk of severe infection and hospitalization, particularly in the presence of pulmonary disease as found in our study (10, 26). Early treatment, with higher than physiologic doses (or stress doses) of glucocorticoid, is warranted for patients with AI at the onset of COVID-19 symptoms.

We also found that mean supraphysiologic exogenous doses of maintenance glucocorticoids prior to contracting COVID-19 correlated with a higher rate of COVID-19^+ve^ infection. After removing individuals using doses over 60 mg daily (*n*=20), there was no difference among the three groups. Prophylaxis with higher doses of glucocorticoids may increase infection risk. Maintaining physiologic dose that mimics normal cortisol production and avoiding periods of relative adrenal insufficiency is recommended.

A multivariate model of rheumatoid arthritis revealed a > 2-fold risk of severe COVID- 19^+ve^, or death, for patients taking TNF inhibitors plus prednisone, compared with those on conventional disease modifying drugs or non-rheumatoid arthritis controls (28). Similar comorbidities among patients with hypoadrenalism and rheumatoid arthritis render both at enhanced susceptibility to COVID-19 (28).

Interruption in maintenance therapy in 2020 occurred for 10% of respondents and 12.5% of those testing COVID-19^+ve^. These patients are at-risk for an Addisonian crisis in addition to COVID-19, underscoring the need for an emergency kit and uninterrupted supply of glucocorticoids.

In addition to AI, we found older age (40–60 years), as an independent risk factor, was associated with a higher risk of contracting COVID-19. The literature supports comorbid disease along with advancing age, obesity, diabetes, lung, cardiac and cardiovascular disease (CV) have been associated with higher risk (29, 30). We did not interrogate BMI and although higher levels of CV disease were reported, these were not statistically significant predictors of COVID-19 risk. Exposure risk related to lower frequency of mask use and social distancing was associated with higher frequency of infection, reinforcing the need for self-protective behaviors.

Data at the inception of the pandemic, demonstrated a higher predilection for malescontracting COVID-19 and a 3 fold likelihood of requiring ICU admission with a higher mortality risk (31). Thus, we explored the incidence for males separately. We found male CAH patients at higher risk. It is possible in male patients with CAH, high concentrations of androgens independently suppress immunity and may enhance SARS CoV-2 viral spike binding to ACE2 receptors in the adrenals and testes thereby reducing antigen recognition in the host cell (32). Although our findings accord with the aforementioned data, the relatively small size of respondents with COVID-19, the underrepresentation of males, and the possibility of a type 1 error, demands any conclusions to be considered with caution.

Pulmonary disease was predictive of infection and hospitalization with more severe symptoms regardless of AI etiology. Loss of protective NK cell function is reported in the context of recurrent lung infections, possibly enhancing the risk of infection (10, 26, 27). We did not interrogate smoking, which has been linked to more severe COVID-19. Inhaled steroids for pre-existing asthma and a history of chronic obstructive pulmonary disease and obstructive sleep apnea (diagnosed and undiagnosed) have been reported to increase disease severity (33, 34). For patients with AI, standard mortality ratios are 2.2-8.9-fold higher than the general population, and often as the result of a trivial respiratory infection and impaired innate immunity (35, 36). Respiratory distress was the precipitant for hospitalization in 55.5% of our respondents. ICU admission and ventilator support were reported in only 2/9 (22%) of COVID- 19^+ve^ hospitalized respondents, both of whom reported pre-existing pulmonary dysfunction.

Post viral syndromes are reported after initial recovery from COVID-19 infection (37). Persistent low energy, chronic fatigue, low mood and dizziness were reported, despite physiologic steroid replacement doses.

This is a large multi-national, multilingual survey of patients with AI with COVID-19 status, which was strengthened by large size and global representation, likely improving its generalizability. Multiple AI etiologies were included and assessed independently, whereas missing data were avoided in the survey design with no response interpretation required. It is acknowledged that numbers of untested patient may include asymptomatic patients or poor access to testing, in which case the incidence of hospitalization would be reduced.

Criticisms of the study design may arise, invoking possible bias from its exclusive internet methodology and exclusion of those without access or who are technically challenged. However, 59.5% of the global population are active internet users which is intriguingly greater than the WHO estimates of fewer than 50% who have access to medical care (www.statistica.com). The use of multiple platforms can minimize bias. Counter to this, our study design avoided face to face transcription and interpretation error. The survey was developed using recommended principles of survey research, including: objective driven, pre- tested, voluntary and widely distributed (38).

Limitations include potential self-selection bias, inability to verify diagnosis and recall bias. We believe that several factors help to minimize these limitations including the wide distribution and the size of the sample in this study and the current emphasis on the patient’s disease knowledge, empowerment and need for self-management (4, 6, 7). Direct access to ones own electronic medical records that contain diagnosis and comorbid conditions, plus the availability of medical information on the internet, has resulted in a much more, well informed patient population, regardless of diagnosis. The mortality risk associated with adrenal crisis and COVID-19 are both potential motivators for disease knowledge. However, interrogation of hospital records for mortality data is recommended for future studies. Stress dosing of glucocorticoids during infection is paramount for patient with AI. Although stress dosing was reported in this survey, efficacy in the context of COVID-19 was not evaluated. Interrogation of hospital records is recommended for this purpose in future studies. Likewise regional prevalence of AI and incidence of COVID-19 is not reflected in this data and is recommended for future analysis.

## Conclusion

We report a substantially higher incidence of COVID-19 and hospitalization in patients with AI globally compared to the background COVID-19 population. Additionally, premorbid chronic lung conditions, higher maintenance doses of glucocorticoids, a diagnosis of CAH and male sex, were associated with COVID-19. This data supports the need for an uninterrupted supply of glucocorticoids, physiologic maintenance doses, early stress glucocorticoids for acute symptoms of COVID-19, and emphasizes the importance of behavioral protective measures for patients with adrenal insufficiency independent of etiology. Futher studies are recommended to evaluate risk in males with CAH, efficacy of stress steroids in the context of COVID-19 infection and comparison of regional AI prevalence and COVID-19 incidence.

## Data availability statement

The raw data supporting the conclusions of this article will be made available by the authors, without undue reservation.

## Ethics statement

Ethical review and approval was not required for the study on human participants in accordance with the local legislation and institutional requirements. Written informed consent to participate in this study was provided by the participants’ legal guardian/next of kin.

## Author contributions

All authors listed have made a substantial, direct, and intellectual contribution to the work and approved it for publication.

## Acknowledgments

The authors thank Shirley McCartney, PhD (Oregon Health & Science University) for editorial assistance.

## Conflict of interest

The authors declare that the research was conducted in the absence of any commercial or financial relationships that could be construed as a potential conflict of interest.

## Publisher’s note

All claims expressed in this article are solely those of the authors and do not necessarily represent those of their affiliated organizations, or those of the publisher, the editors and the reviewers. Any product that may be evaluated in this article, or claim that may be made by its manufacturer, is not guaranteed or endorsed by the publisher.
